# A new species group of *Strumigenys* (Hymenoptera, Formicidae) from Ecuador, with a description of its mandible morphology

**DOI:** 10.3897/zookeys.1036.62034

**Published:** 2021-05-05

**Authors:** Douglas B. Booher, Philipp O. Hoenle

**Affiliations:** 1 Yale Center for Biodiversity and Global Change, 165 Prospect Street, New Haven, CT 06520-8106, USA Yale Center for Biodiversity and Global Change New Haven United States of America; 2 Georgia Museum of Natural History, 101 Cedar Street, Athens, GA 30602, USA Georgia Museum of Natural History Athens United States of America; 3 Ecological Networks, Department of Biology, Technical University of Darmstadt, Darmstadt, Germany Ecological Networks, Department of Biology, Technical University of Darmstadt Darmstadt Germany

**Keywords:** 3D scan, µCT, LaMSA (latch-mediated spring-actuation), Myrmicinae, Northwest Ecuador, power amplified, *Strumigenys ayersthey*, taxonomy, tropical forest

## Abstract

*Strumigenys* is one of the most diverse ant genera in the world and arguably the most morphologically diverse, exhibiting an exceptional range of mandible shape and function. A new species, *Strumigenys ayersthey***sp. nov.**, discovered in the Chocó region of Ecuador is described. With two morphological characters, this species is shown to be a morphologically unique outlier among *Strumigenys* globally, having predominately smooth and shining cuticle surface sculpturing and long trap-jaw mandibles. Using µCT scans, we produced 3D images of the worker ant and static images to examine and compare mandible articular morphologies with most morphologically similar members of the *mandibularis* species group. Cuticular, pilosity, and articular mandible morphological differences supports placing the new species in its own new species group.

## Introduction

Ecuador has one of the highest animal and plant species richness of any country, both in terms in of species per area and total species richness (Sierra et al. 2002). This unusually high diversity is due to the three very distinct bioregions within Ecuador: the Amazon basin in eastern Ecuador, the Chocó-Darién bioregion in the northwest, and the Tumbesian drylands in the southern portion of the country (Sierra et al. 2002). Of these, the areas west of the Andes have been the least studied, and particularly the Chocó-Darién is a hotspot for new, previously unknown ant species (Donoso and Ramón 2009; Donoso et al. 2009; Salazar and Donoso 2013; Salazar et al. 2015; Donoso 2017; Hoenle et al. 2020). The *Strumigenys* fauna of Ecuador currently includes 51 species (Salazar et al. 2015), several of which are endemic (e.g., *Strumigenys madrigalae*Lattke and Aguirre 2015). Here, we report the finding of another likely endemic *Strumigenys* species from the Ecuadorian Chocó, contributing to a better understanding of this hyperdiverse region.

*Strumigenys* is one of the most diverse ant genera known with currently 852 extant and four fossil species, and is present on all continents except Antarctica (Guénard et al. 2017; Bolton 2020). Over the past two decades this genus received much taxonomic attention, but given the number of recent species descriptions, it is certain that many species are still waiting to be discovered (e.g., Booher et al. 2019; Sarnat et al. 2019; Dong and Kim 2020). *Strumigenys* are comparatively small ants (most < 4 mm) and are primarily litter dwelling although there are a few arboreal species (Bolton 2000). Most species assessed for diet are specialist predators of entomobryomorph Collembola (springtails), which may have led them to evolve a range of peculiar mandible forms to facilitate predation of fast-moving prey (Wesson and Wesson 1939; Wilson 1953; Masuko 1984; Dejean 1985; Brown and Wilson 1995; Masuko 2009; Lattke et al. 2018; Gray et al. 2019; Booher et al. 2021). Most spectacular, many *Strumigenys* possess trap-jaws, fast-snapping mandibles that function via a power amplified latch-mediated spring-actuation (LaMSA) (Booher et al. 2021; Ilton et al. 2018; Longo et al. 2019) akin to a biological mousetrap (Gronenberg 1996; Larabee and Suarez 2014). Performance and evolution of the trigger and latch system has been studied in detail, however there has been little attention given to additional undefined mandibular morphology that may contribute to the stability of trap-jaw movement in *Strumigenys* and other trap-jaw ants (Gronenberg 1996; Larabee et al. 2018; Booher et al. 2021). Within *Strumigenys*, the LaMSA mechanism has evolved independently multiple times, with each evolution convergent in morphology, function, and performance (Booher et al. 2021). However, the morphological variation in articular surfaces and articular processes involved in mandible movement across *Strumigenys* with or without LaMSA is morphologically variable and not well understood (Booher, unpublished data, Silva and Feitosa 2019). Here, we construct and define the single species *ayersthey* species group, describe the mandible articular morphology in detail within the description of the previously unknown *S. ayersthey* sp. nov., and compare it to that of morphologically similar members of the *S. mandibularis* species group to support species group separation.

## Materials and methods

### Sampling and geographic origins

**Figure 1.**
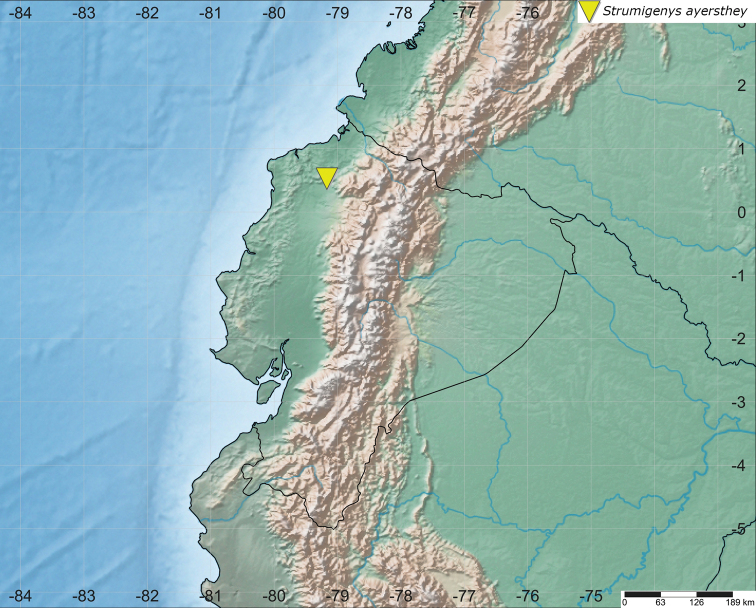
Location of the holotype collection of *S. ayersthey* sp. nov. in Ecuador (Reserva Río Canandé, Esmeraldas Province). Distribution map generated with SimpleMappr (Shorthouse 2010).

The specimen of *Strumigenys ayersthey* sp. nov. was collected during a field trip to the Reserva Río Canandé in Ecuador (Esmeraldas Province) on 2 May 2018 (Fig. 1.). The reserve belongs to the Chocó-Darién bioregion, and is characterized by evergreen tropical forest with a wet season from January to March, and a dry season from September to December. The reserve contains low- to mid-elevation forest spanning a range of approximately 200 to 600 m. The specimen was collected in old-growth forest, along the ridge of small plateau at 507 m elevation. The specimen was collected alive by hand, and later preserved in a vial containing 96% ethanol. The Ministerio de Ambiente de Ecuador issued the permits for collection (MAE-DNB-CM-2017-0068) and exportation (41-2018-EXP-CM-FAU-DNB/MA).

### Photographs

We took stacking images with a Canon EOS 7D with a MPE 65mm lens (Canon, Tokyo, Japan). We used Helicon Focus Version 7 (Helicon Soft Ltd., Kharkiv, Ukraine) to focus stack multiple images, and added a scale and brightness adjustments with Adobe Photoshop CS6 13.0 (Adobe Inc., San Kaso, CA, USA). All images presented are available online and can be viewed on AntWeb (Antweb 2020), where it can be identified by a specimen-level code affixed to the pin.

### Synchrotron X-Ray micro-computed-tomography (SRµCT) scan

The SRµCT scan of the sample was recorded at P05 at PETRA III, Deutsches Elektronen-Synchrotron DESY in Hamburg, Germany. We used absorption contrast tomography with an energy of 11 keV, a sample-detector distance of 20 mm, and a magnification of 9.97 resulting in an effective pixel size of 0.642 µm.

The dataset has been cropped, positioned, and visualized in VGStudio MAX 3.0 (build 109953; Volume Graphics GmbH, Heidelberg, Germany). Amira 5.6 (FEI Visualization Sciences Group, Mérignac Cedex, France) was used to digitally remove the cardboard the specimen was glued onto and to make a surface model of the scan data. Fiji (Schindelin et al. 2012) was then used to convert the resulting surface model to U3D.

### Morphological data

The measurements, indices, and morphological terminology used in species-group definitions and species descriptions in this study are based on Bolton (2000), and the mandible articular terminology is based on two studies (Table 1) (Silva and Feitosa 2019; Richter et al. 2019). We compared anologous terms for these studies, and added our own terminology for features that were not included in these studies in best agreement with terminology already in use. Measurements were taken using the measurement application of the LAS-X Leica software using a Leica IC90 E digital camera and Leica M165 C microscope with either a 1.0× or 1.6× PLANAPO objective. Measurements and indices are presented as a single value mean of three independent measures; measurements are expressed in millimeters to three decimal places. Global morphological mandible index data were assimilated by DBB (Booher et al. 2021). Specimens were identified without head surface sculpture visually from species imaged and hosted on AntWeb (Antweb 2020). The data was plotted with JMP version 15.0.0 statistical software. Softening specimens and visual confirmation of trap-jaw mechanisms through visual manipulations of specimen were done as described in Booher et al. (2020). For this study, we examined mandible morphology in the following *mandibularis*-group species: *S. planeti*CASENT0873025, *S. biolleyi*CASENT0747760, *S. cordovensis*CASENT0609666, and *S. smithi* from Ecuador in author DB’s collection.

**Table 1.** Comparison of morphological features of *Strumigenys ayersthey* sp. nov. with those described in *Strumigenys* spp. (Silva and Feitosa 2019), and the Myrmicine ant *Wasmannia affinis* (Richter et al. 2019). *Strumigenys ayersthey* sp. nov. has several features previously not reported, but may be shared with many other *Strumigenys*. Presence refers to the reporting of each morphological feature: S – *Strumigenys* including *S. ayersthey*, SA – only reported in this publication in *S. ayersthey*, W – reported in *Wasmannia affinis*.

Abbreviation			Presence	Definition	Figure
This study	Silva and Feitosa 2019	Richter et al. 2019			
aba	NA	apab	SA&W	apodeme attachment location of the abductor muscle	Fig. 6
ada	NA	apad	SA&W	apodeme attachment location of the adductor muscle	Fig. 6
clp	clp	cl	S&W	clypeus	Fig. 5
dfc	NA	dma (of head)	SA&W	dorsal mandibular articular surface of clypeus	Fig. 5
dmap	dmap	dma (of mandible)	S&W	dorsal articular process of mandible	Figs 5, 6
lbp	lplb	lbrp	S&W	labral articular process	Figs 5, 6
lbh	NA	NA	SA	labral hood of basal mandibular process insertion	Fig. 5
lbm	labrum	lbr	S&W	labrum	Fig. 5
lmap	lmap	abs (abductor swelling)	S&W	lateral articular process of mandible	Figs 5, 6
md	mandible	mandible	S&W	mandible	Fig. 5
vmap	vmap	vma (of mandible)	S&W	ventral articular process of mandible	Figs 5, 6
vpc	NA	NA	SA	ventral articular process of clypeus	Fig. 5
lmah	NA	absa (of head)	S&W	articular area of the abductor swelling	NA
vmah	NA	vma (of head)	S&W	ventral mandibular articulation	NA
bpm	bpm	NA	S	basal process of mandible	Figs 5, 6

### Measurement definitions

**CI** Cephalic index. HW/HL × 100;

**EL** Eye length. Maximum length of eye as measured in oblique view of the head to show full surface of eye;

**FL** Femur length. Maximum length of hind femur;

**HL** Head length. Maximum length of head in full-face view, excluding mandibles, measured from anterior most point of clypeal margin to midpoint of a line across the posterior margin;

**HW** Head width. Maximum width of head in full-face view, measured in the same plane as HL;

**MI** Mandible index. ML/HL × 100;

**ML** Mandible length. The straight-line length of mandible at full closure, measured in the same plane as HL, from mandibular apex to anterior clypeal margin;

**PW** Pronotum width. Maximum width of pronotum in dorsal view;

**SI** Scape index. SL/HW × 100;

**SL** Scape length. Length of antennal scape excluding the basal condylar bulb;

**TL** Total body length;

**WL** Weber’s Length.

## Results

### Key to *Strumigenys ayersthey* sp. nov.

**Table:** 

1	Head in full face view absent of sculpture, smooth and shining; mandible relatively long MI 65; pilosity consisting of nearly uniform sub-erect to erect filiform setae	***Strumigenys ayersthey* sp. nov. (Ecuador)**
–	Head in full face view usually with at least some sculpture, if smooth and shining; mandible is relatively short MI < 40; pilosity variable but not usually consisting of nearly uniform sub-erect to erect filiform setae	**Couplet 1 in Bolton (2000; Key to Nearctic and Neotropical *Strumigenys*)**

### *Strumigenys ayersthey* group

**Figure 2.**
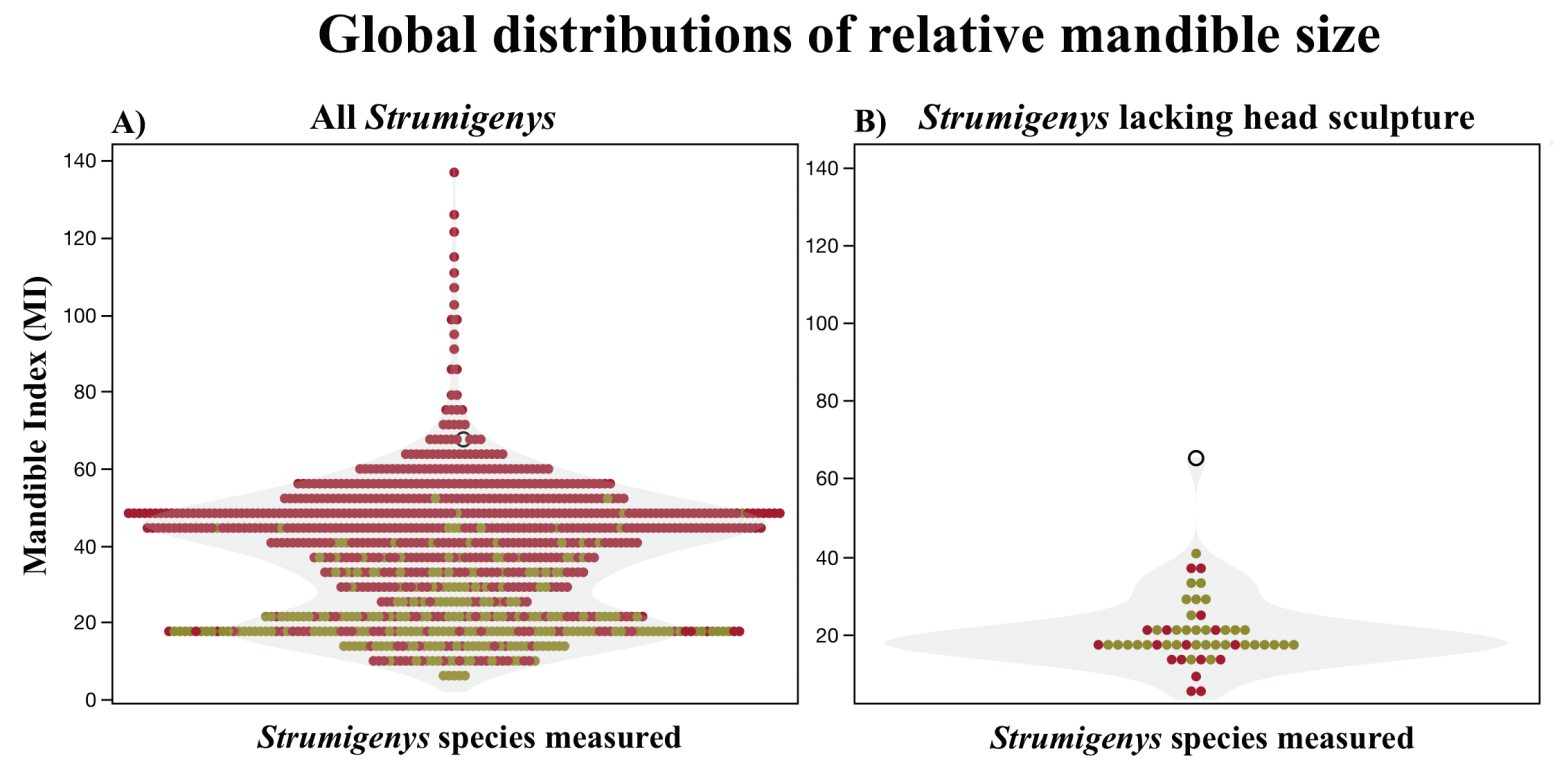
Comparisons of MI among *Strumigenys* spp. **A** accounts of 961 species and morphospecies globally representing all species groups **B**MI of 52 *Strumigenys* identified as not smooth and shining cuticular surface of the head in full frontal view. Light yellow points are species without trap-jaws, dark red points are those with trap-jaws. *Strumigenys ayersthey* sp. nov. is marked with an open black circle and possesses trap-jaw mandible morphology.

The *ayersthey* group contains one member and exhibits most morphological resemblance to the *mandibularis* group (Bolton 2000), from which it is most easily separated by differences in sculpture and pilosity. *Strumigenys ayersthey* sp. nov. has little to no sculpture anywhere on its body and has only fine simple to flagellate setae, whereas *mandibularis* species group members are predominately sculptured and not shining with mostly decumbent to appressed apically expanded or flattened setae. Also separating these two groups are morphological differences in dorsal articular processes of mandibles, in *S. ayersthey* sp. nov. these processes project from the dorsal surface at the base of each mandible without distinct lamellate lateral edges. In *mandibularis* species-group members, these processes arise from laterally expanded lamella at the base of mandibles that are continuous with the dorsal surface of each mandible. *Strumigenys ayersthey* sp. nov. can be distinguished from all other *Strumigenys* species by shining sculpture, MI 65, and ML 41, other *Strumigenys* predominately lacking sculpture and shining have MI < 40 and ML < 0.25 (Fig. 2). The following diagnosis is adapted and expanded from the *mandibularis* species-group diagnosis (Bolton 2000).

### *Strumigenys ayersthey* species group: diagnosis of worker.

- Bulla of femoral gland not easily visible but appears as a faint streak along the medial dorsal surface.
- Scape not dorsally flattened.
- Apical fork of mandible with one well-developed intercalary tooth. Mandible with two conspicuous acute preapical teeth; both approximately the same length. Preapical dentition not crowded near apex. MI 65.
- Anterior clypeal margin usually shallowly convex.
- Leading edge of scape usually with all setae standing and directed toward apex of the scape. Scape slender, the subbasal curve extremely shallow; relatively long, SI 110.
- Preocular carina in profile short, terminating before level of eye.
- Upper margin of the antennal scrobe not sharply defined behind level of eye.
- Ventrolateral margin of head continuous and not obviously concave in front of eye.
- Postbucal impression absent.
- Propodeum with minute teeth with a lower propodeal tooth-like lobe at base of declivity that is slightly less developed than the upper propodeal tooth, the two linked by a lamella.
- Ventral surface of petiole with spongiform tissue.
- Pilosity. Pronotal humeral setae flagellate and indistinguishable from neighboring background pilosity of similarly shaped simple standing to flagellate setae. Standing setae on head and mesosoma not differentiated from ground pilosity, abundant and simple to flagellate.
- Sculpture. Head and mesosoma predominantly or entirely free of sculpture and shining, usually with a smooth area on mesopleuron.
- Basal process of mandible arises dorsally with a locking angle estimated between 180 and 200°.
- Dorsal articular process of mandibles bluntly pointed arising evenly from the dorsal surface without a distinct lateral lamella.
- Basal mandibular process arising in dorsal most plane of mandibles.
- Processes of clypeus present as a pair of small tooth like laminar ridges each positioned between the basal mandibular and dorsal articular processes of mandibles in closed position.

#### Strumigenys ayersthey sp. nov.

Taxon classificationAnimaliaHymenopteraFormicidae

976447E8-F287-5FA8-97E3-E604541C9A9B

http://zoobank.org/235F1F9D-A33F-4C75-959F-C52B9BC5FD41

##### Type material examined.

***Holotype worker*: Ecuador: Esmeraldas Province**, Reserva Río Canandé, 2 May 2018, Elevation 507m, 0.5263, -79.1682, Part of diversity study Hoenle & Blüthgen plot F1N31, hand-sampling on forest floor in primary forest, specimen broke in several parts, leg. P. Hoenle. Specimen identifier code (casent0875770), deposited at [MEPN] (Museo de Colecciones Biológicas Gustavo Orcés, Escuela Politécnica Nacional, Quito, Ecuador).

**Figure 3.**
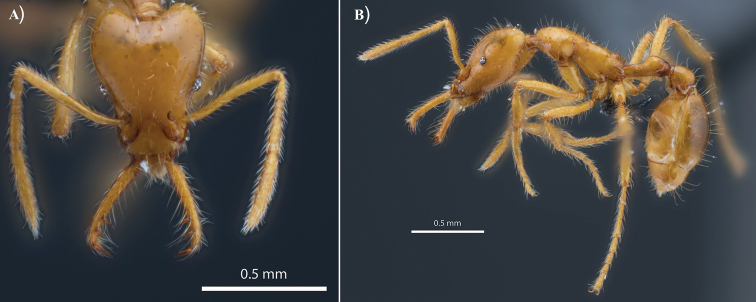
Images of **A** head in full-face view and **B** profile of Holotype specimen of *Strumigenys ayersthey* sp. nov. (CASENT0875770) [MEPN].

##### Holotype worker measurements

(n = 1): HL = (0.609); HW = (0.480); ML = (left = 0.383, right = 0.411), the left mandible is slightly shorter than the right mandible; PW = (0.303); SL = (0.530); FL = (0.568); EL = (0.07); WL = (0.683); CI = (78.82); MI = (65.19); SI = (110.42).

**Figure 4.**
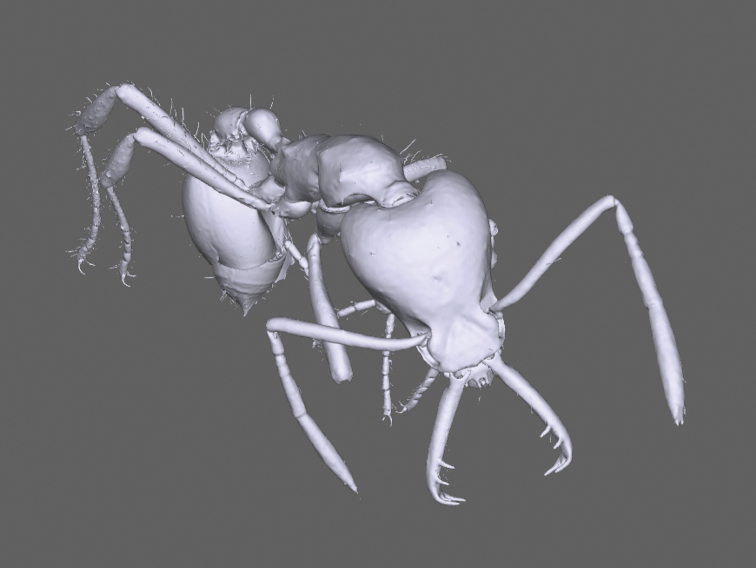
3D scan of *Strumigenys ayersthey* sp. nov. assembled by µCT.

##### Description.

**Figure 5.**
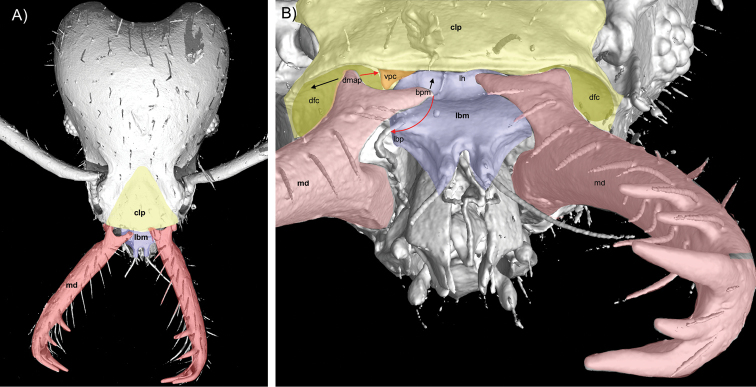
Colorized µCT surface renders of the head of *S. ayersthey* sp. nov. **A** head in full face view and **B** view from apex of mandibles looking towards base of mandibles. Black arrows represent closing motions and red arrows represent opening motions of mandibles. Abbreviations: **bpm** – basal process of mandible, **clp** – clypeus (yellow), **dfc** – dorsal articular surface of oral cavity (green), **dmap** – dorsal articular process of mandible, **lbp** – labral articular process, **lh** – labral hood, **lbm** – labrum (lavender), **md** – mandible (red), **vmap** – ventral articular process of mandible, **vpc** – ventral articular process of clypeus in orange. As the mandibles open towards latched position, the labrum (lbm) hinges upwards such that the basal mandibular process (bmp) latches into the complementary pocket of the labrum (lbp) and the dorsal articular process of the mandible (dmap) articulates freely within the dorsal articular surface of the oral cavity (dfc) around the ventral process of the clypeus (vpc). The labral hood (lh) and the ventral processes of the clypeus (vpc) forms a pair of pockets housing the basal mandibular process (bmp) of each mandible.

**Figure 6.**
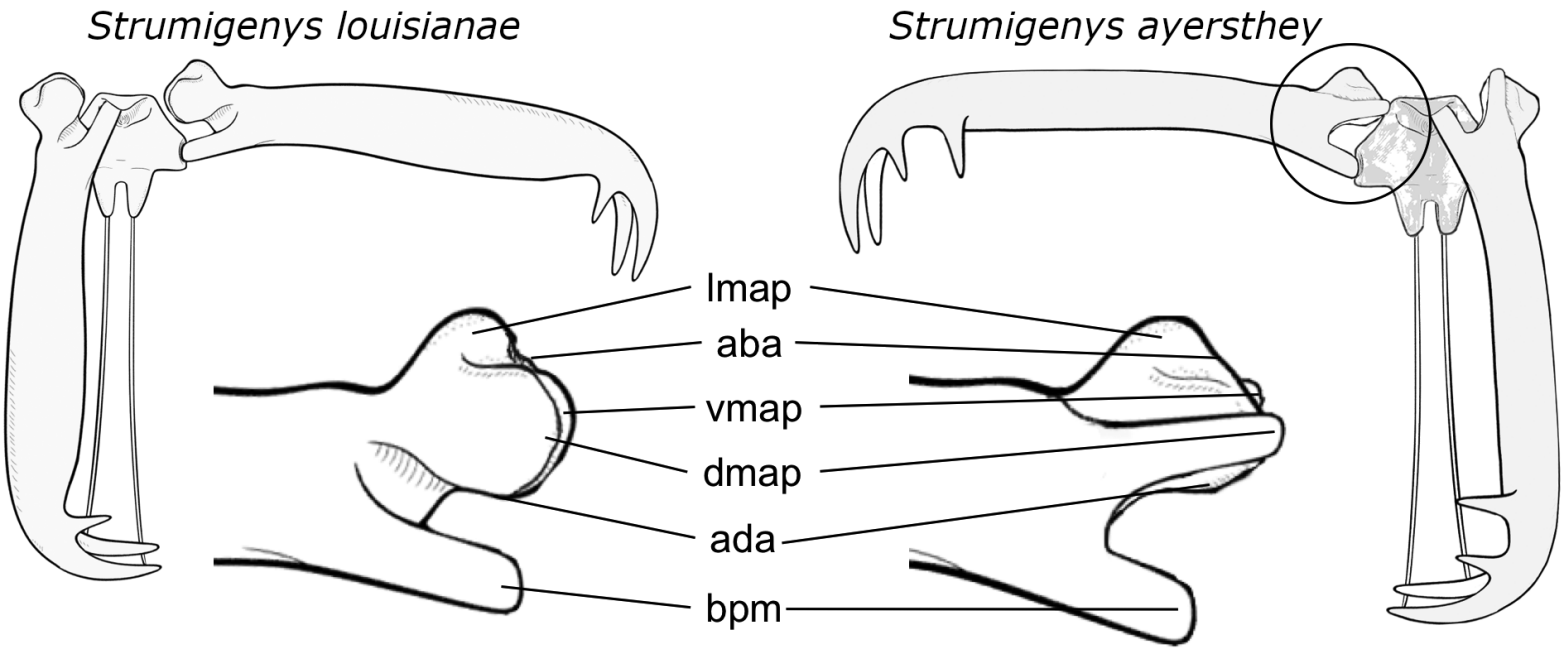
Comparison of the mandibles between *S. louisianae* (left) and *S. ayersthey* sp. nov. (right). Abbreviations: **aba** – apodeme attachment location of the abductor muscle, **ada** – apodeme attachment location of the adductor muscle, **bpm** – basal process of mandible, **dmap** – dorsal articular process of mandible, **lmap** – lateral articular process of mandible, **vmap** – ventral articular process of mandible. Illustrations adapted from Booher et al. (2021).

***Mandibles*** with five teeth; two preapical teeth, apicodorsal and apicoventral teeth, and an intercalary tooth. The two preapical teeth are well developed and spiniform with nearly equal lengths and are longer than the width of the mandible where they arise (first preapical tooth = 0.056, second preapical tooth = 0.050). These teeth are located in the apical third of mandible and separated by a distance approximately equal their length (0.051). Apicodorsal (0.78) and apicoventral (0.73) teeth spiniform and of nearly equal length and with a well-developed intercalary tooth (0.38) arising just above the apicoventral tooth. Basal portion of mandible with four processes, three articular processes (dorsal, lateral, and ventral articular processes) and a latching process (basal mandibular process; Fig. 5). The dorsal articular process extends posteriorly from the basal dorsal surface without a distinct lateral ridge and terminating as a small bulbous point. The ventral articular process extends from the latero-posterior basal portion of the mandible as a dorsal to ventral cuticular ridge from and is continuously connected to the lateral articular process. The lateral articular process is dilated, with the medial portion extending laterally away from a line drawn vertically from the posterior-most positions of the dorsal and ventral processes. In full face view, the lateral articular process appears as a lateral bulge below the dorsal ridge of the dorsal articular process and shadows the ventral process (Fig. 6). The dorsal area between the basal process and dorsal articular process is indented and when mandibles are closed the process of the clypeus extends into this cavity (Fig. 5).

***Clypeus*** ca. 1.5 × as wide as long. Eye apparent (0.070) with 15 or 16 pigmented ommatidia. Scape sub-cylindrical with shallowly curved subbasal bend. Ventrolateral margin of head in front of eye not sharply defined, strongly indented or concave. Postbuccal impression absent. Preocular carina and upper margin of the antennal scrobe in profile short, terminating anterior of eye.

***Mesosoma*** shallowly and gradually impressed between pronotum and propodeum. Declivity of propodeum with two bluntly rounded triangular teeth that are just longer than the lamella connecting them (upper tooth = 0.062, lower tooth = 0.50, lamella at shallowest point between = 0.046).

In profile view, bulla of propodeal spiracle located at dorsal-most position of propodeum with propodeal spiracle opening facing postero-dorsally and forming lateral bulges that disrupt the outline in dorsal view. Spiracle opening much narrower than EL (.022). Petiolar node longer (0.127) than wide (0.113). Postpetiolar disc longer (0.185) than wide (0.153.). First gastral tergite with no basigastral costulae past the limbus.

***Sculpture*.** Head and rest of body smooth and shining and without obvious sculpture other than piliferous punctations where setae arise. Basigastral sculpture limited to costulae within the limbus and do not extend onto the surface of the first gastral tergite.

***Pilosity*.** The background pilosity of all surfaces (mandibles, head, mesosoma, petiole, postpetiole, abdomen, and legs) are covered in evenly spaced simple to subflagellate erect to suberect setae that vary in length and are apically pointed. Head without differentiated apicoscrobal setae and leading edge of scape also without differentiated setae, pilosity of scape on all surfaces consists of short erect simple setae tending to point towards apex, none are recurved as to point to the base, and scape pilosity is similar to those elsewhere on head. Differentiated longer subflagellate to flagellate setae are limited to a pair straddling the midline on the anterior margin of clypeus that extend over mandibles when closed, a lateral pair on pronotal shoulders, a pair arising from ventral portion of propodeal spiracle, one to two pairs on the dorsum of petiole, and postpetiole. The majority of pilosity on gaster consist of slightly longer subflagellate setae than those on mesosoma.

***Spongiform appendages*.** Length of lateral lobe of petiole weakly developed and visible only as a thin carinae along posterior third of node; expanded as a thin cuticular flange just behind the node in dorsal view. Subpetiolar flange developed as a thin cuticular narrow flange deepest posteriorly (0.046). Lateral lobes of postpetiole distinct and separated from the anterior flange of the post petiolar disc and do not connect posteriorly leaving a medial posterior gap along the posterior portion of disc (most easily seen in dorsal view). In profile, ventral lobe of postpetiole also weakly-developed (0.053 in depth) and much narrower than the exposed height of postpetiolar node (0.149).

***Color*.** Yellow uniform light reddish brown.

##### Queen and male.

Unknown.

##### Etymology.

Many cultures have recognized a spectrum of genders between and beyond the binary of male and female. However, by following a rule exampled in the International Code of Nomenclature (ICZN 1999) for how to name species after individuals, one might conclude only binary gender assignments possible when assigning new species names derived from Latin. Dubois (2007) provides clarification to this rule stating that there is no need to amend or Latinize personal names – and therefore no need to assign gender. In contrast to the traditional naming practices that identify individuals as one of two distinct genders, we have chosen a non-Latinized portmanteau honoring the artist Jeremy Ayers and representing people that do not identify with conventional binary gender assignments, *Strumigenys ayersthey*. The ‘they’ recognizes non-binary gender identifiers in order to reflect recent evolution in English pronoun use - ‘they, them, their’ and address a more inclusive and expansive understanding of non-neutral gender identification. *Strumigenys ayersthey* sp. nov. is thus inclusively named in honor of Jeremy Ayers for the multitude of humans among the spectrum of gender who have been unrepresented under traditional naming practices. Jeremy was a multifaceted and beloved Athens-based (GA, USA) artist and activist whose humanity and achievements defied the limits of categorized classification. Jeremy brought an intellectual and playful, Pan-like curiosity to every aspect of his life. He was a writer, philosopher, painter, musician, activist, photographer, gardener, and exploder of boundaries who transformed the culture that surrounded him. His deep appreciation of the variety and minute details of the natural world astounded all who knew him. In the spirit of Jeremy, we also propose that the -they suffix can be used for singular honorific names of non-binary identifiers in compliance with the ICZN.

## Discussion

As morphological convergence is rampant among *Strumigenys* morphotypes (short or long mandible species) it is difficult to determine by morphology alone how species are related (Ward et al. 2014). However, within biogeographic regions, species groups of morphologically similar *Strumigenys* species are often phylogenetically most closely related (Booher 2021). As such, morphological species groups are relevant and useful for identification as well as evolutionary research (Booher et al. 2021). In the construction of *Strumigenys* morphological species groups, differences in the position, presence, and shape of pilosity are of major importance. For example, the direction and shape of hairs along the clypeal margin and along the leading edge of the scape separates several Nearctic species groups, e.g., *pulchella*, *ornata*, and *talpa* groups (Bolton 2000). Similarly, slight differences in sculpturing help to identify similar species, but major differences in sculpture (i.e., having sculpture present across most cuticular surfaces compared to no sculpture) do not occur among species within any *Strumigenys* species group. We further justify the formation of a new single species group with differences in basal mandibular morphology from most morphologically similar *mandibularis*-group members.

The general mandibular morphology of LaMSA*Strumigenys* has been well described with the base of the mandible having three articular processes; the dorsal and ventral articulatory processes are responsible for holding mandibles in place during movement and a third lateral process is attached via apodemes to opening muscles (Fig. 6) (Silva and Feitosa 2019), alternatively termed the abductor swelling of the mandible or ‘atala’ (Richter et al. 2019; Richter et al. 2020). Although articular morphology of mandibles has been described in LaMSA*Strumigenys* and more generally in the more typical ant genera *Wasmannia* (Richter et al. 2019), *Formica*, and *Brachyponera* (Richter et al. 2020), there has not yet been a comparison in morphological features between them and there are a few important differences associated with the derived morphology of trap-jaws in *Strumigenys* (Table 1). Most morphological features in *S. ayersthey* have homologous features shared with other ants, however there are a few features that are not shared or have not been previously reported and are worth discussing. A most apparent difference is the dorsal articulation of the mandible and head. In what is described in *Wasmannia* and other ants, mandibles are tightly connected to the head capsule with primary and secondary joints, with the “secondary joint (dmah-dmap) formed by a ventrolateral longitudinal smooth elongation of the clypeus… which articulates with a smooth dorsolateral area on the mandibular base” (Richter 2019). In *S. ayersthey* sp. nov., this secondary joint is not connected to the head and the dmap moves freely within the dfc. We hypothesize that in contrast to typical ants, the clypeal articular process present in *S. ayersthey* helps to stabilize dorsal mandible articular movement. A second morphological feature important to note, is the derived labral hood (lh) present in *S. ayersthey*. This dorsal expansion of the basal area of the labrum is highly sclerotized, hypothesized to reduce damage from self-piercing and over-rotation, and is common to trap-jaw *Strumigenys* (Booher et al. 2021). We provide a table of mandible terminology (Table 1), however a more extensive comparative study across ants is needed to truly understand homology of mandible morphology.

Less prominent morphological features differ between trap-jaw *Strumigenys* and, for instance, mandible dentition has been used as focal distinguishing character between species groups. *Strumigenys ayersthey*, although most similar to members of the *mandibularis*-group, the dorsal articular process of the mandible differs in shape with *mandibularis*-group species. In members of *mandibularis* species group the dorsal articular process arises from a laterally extending dorsal surface forming a shelf like lamellate ridge at the basal portion of the mandible. In dorsal view, this lamellate process overhangs the lateral articular process obscuring most of it from view. In *S. ayersthey* sp. nov., the lateral corner of the dorsal articular surface is gradually rounded and does not form a lamellate margin. Additionally, in the only species with a detached mandible that could be visually inspected by us (*S. planeti*) the posteriormost articular surface of the dorsal process contained three small bulbous points connected by indented lamellae, wherein *S. ayersthey* sp. nov. there is a single bulbous articular point. Therefore, *S. ayersthey* sp. nov. is an exceptional morphological outlier and a rare addition to the hyperdiverse genus *Strumigenys*. It does not fit cleanly into any of Bolton’s species groups, nor can existing species-group definitions envelope this species with minor changes – hence, we placed it as the only member of a new species group. We find morphological articular structure of mandibles are important taxonomic characters and should be investigated in future taxonomic works in this genus.

Our species description includes a µCT 3D render of the holotype worker, and its surface model is freely available for download (Suppl. material 1). This offers any reader virtual morphological details of the new species and the ability to view morphological features at all angles. 3D imaging techniques, and in particular micro-computed X-ray tomography (µCT), are being frequently used in taxonomy and functional morphology, particular in ants (Faulwetter et al. 2013; Akkari et al. 2015; Garcia et al. 2017; Sarnat et al. 2017; Staab et al. 2018). For *Strumigenys*, they already lead to detailed morphological analysis, and µCT scans of Fijian *Strumigenys* have even been suggested as a tool for teaching with augmented reality (Sarnat et al. 2019). In our case, the µCT scan facilitated additional descriptions of mandibular morphology and function of *S. ayersthey* sp. nov.

The discovery of *Strumigenys ayersthey* sp. nov. advanced our understanding of the global morphology of this genus: It’s unique combination of almost no surface sculpturing and long trap-jaw mandibles make it stand out among nearly a thousand other *Strumigenys* species. Because of *S. ayersthey* sp. nov. unusual morphology, information about its general biology could prove to be valuable. However, subsequent attempts in obtaining more specimens at the previous location with Winkler traps in 2019 have failed, and a large ecological ant study in the Canandé reserve did not reveal any more specimens. *Strumigenys ayersthey* sp. nov. can therefore be considered as rare. The discovery of such an unusual rare ant highlights the importance of scientific exploration and conservation of the Chocó region in Ecuador, which is at the same time one of the most biodiverse and threatened areas on our planet (Dinerstein et al. 1995; Olson and Dinerstein 1998; Myers et al. 2000).

## Supplementary Material

XML Treatment for Strumigenys ayersthey

## Supplementary materials

Supplementary material 1 *Strumigenys ayersthey* 3D pdf Data type 3D PDF file Explanation note 3D surface render of *Strumigenys ayersthey*. This is the same file as Figure 4. https://binary.pensoft.net/file/539352This dataset is made available under the Open Database License (http://opendatacommons.org/licenses/odbl/1.0/). The Open Database License (ODbL) is a license agreement intended to allow users to freely share, modify, and use this Dataset while maintaining this same freedom for others, provided that the original source and author(s) are credited.Douglas D. Booher, Philipp O. Hoenle

Supplementary material 2 *Strumigenys ayersthey* apodeme attachments Data type PNG image Explanation note Highlight of *Strumigenys ayersthey* apodome attachments from one slice from the µCT scan. https://binary.pensoft.net/file/539353This dataset is made available under the Open Database License (http://opendatacommons.org/licenses/odbl/1.0/). The Open Database License (ODbL) is a license agreement intended to allow users to freely share, modify, and use this Dataset while maintaining this same freedom for others, provided that the original source and author(s) are credited.Douglas B. Booher, Philipp O. Hoenle
