# Supplementary figures and images for: A new species group of Strumigenys (Hymenoptera, Formicidae) from Ecuador, with a description of its mandible morphology
Source: Zookeys. 2021 May 5;1036:1–19. doi: 10.3897/zookeys.1036.62034 (PMC8116322; doi:10.3897/zookeys.1036.62034)

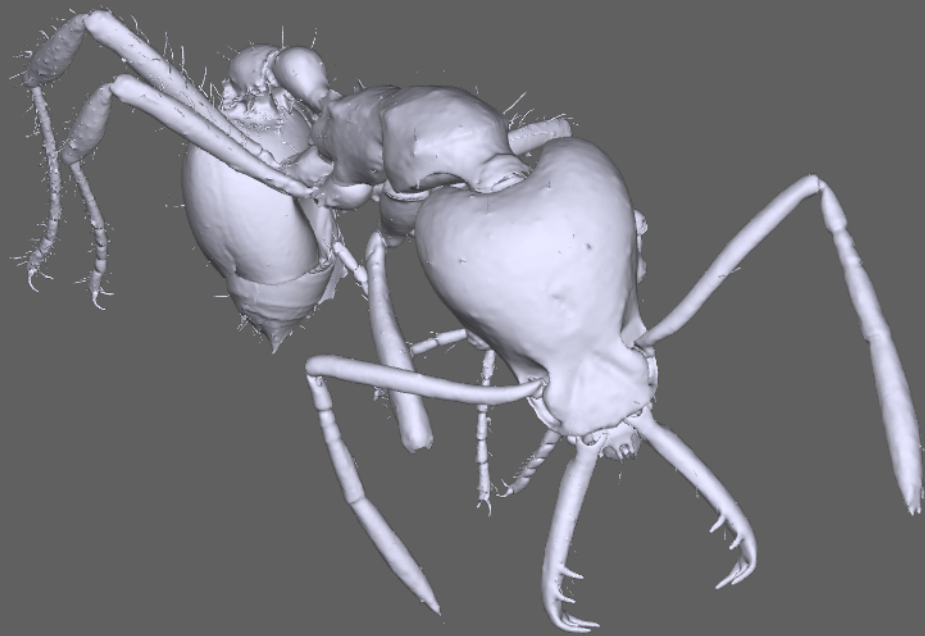

Supplement: Supplementary material 1 — Strumigenys ayersthey 3D pdf [file zookeys-1036-001-s001.pdf]

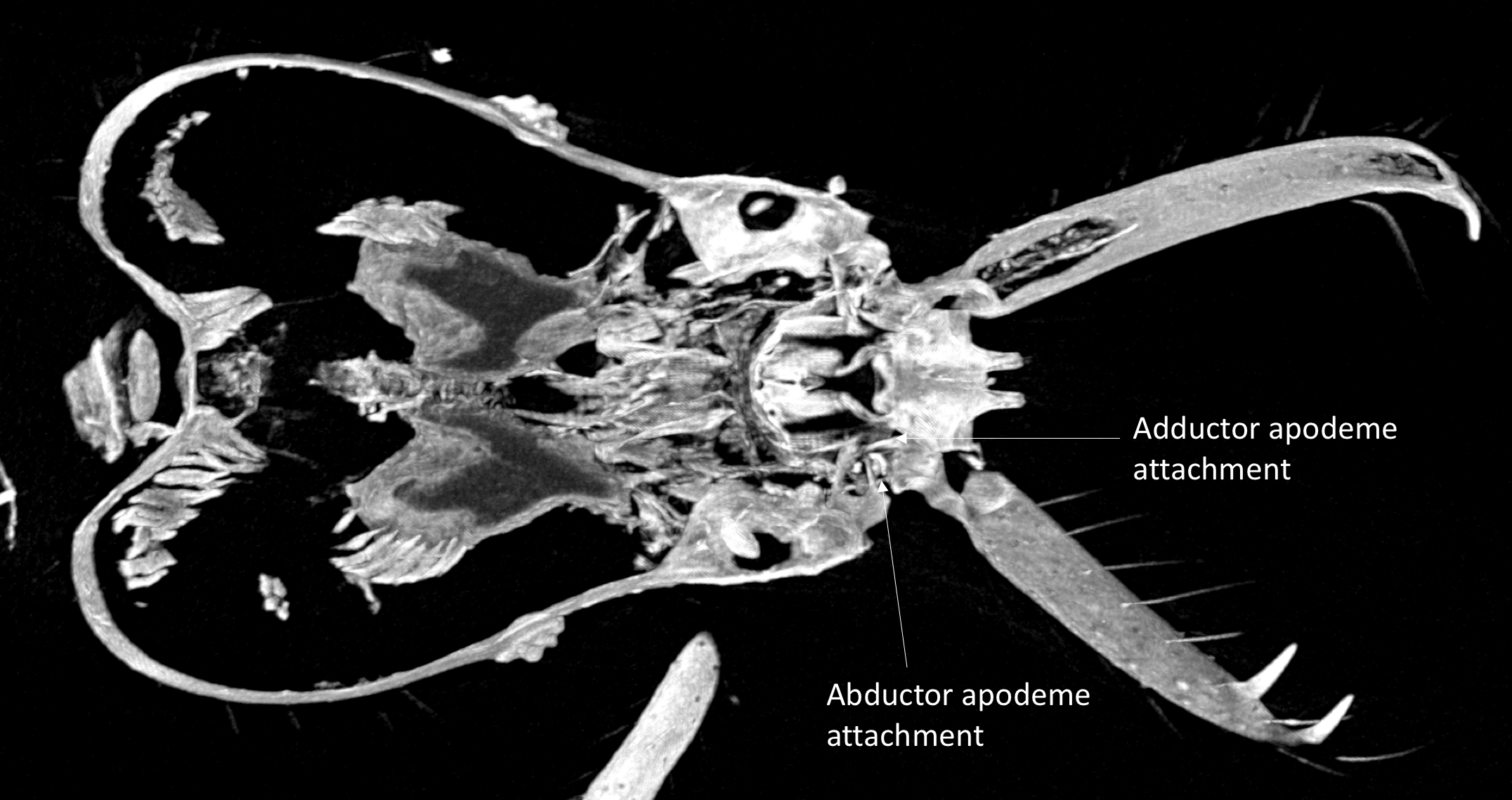

Supplement: Supplementary material 2 — Strumigenys ayersthey apodeme attachments [file zookeys-1036-001-s002.png]
